# The COVID‐19 pandemic and long‐term incentives for developing vaccines: Patent law under stress

**DOI:** 10.1111/jwip.12223

**Published:** 2022-03-31

**Authors:** Mitja Kovac, Lana Rakovec

**Affiliations:** ^1^ Department of Management and Organization, School of Economics and Business University of Ljubljana Ljubljana Slovenia

**Keywords:** COVID‐19 pandemic, incentives, intellectual property rights, law and economics, patent protection

## Abstract

Continents are facing an apocalyptic pandemic that is terribly dangerous for millions of their inhabitants. This paper seeks to address the role of intellectual property (IP) law in addressing the problem of the COVID‐19 pandemic. We suggest that the current international IP law regime and the Trade‐Related Aspects of Intellectual Property Rights Agreement are not insurmountable obstacles for access to a successful COVID‐19 vaccine. The publicly advocated fundamental reform or even abolition of the present IP law regime under serious information asymmetries might be counterproductive and distortive. Via existing compulsory licensing, advance purchase agreements and the employment of patent‐pools, research subsidies, reward mechanisms and reputational sanctions, governments can take the steps needed to effectively overcome any IP‐associated barriers to access to crucial medicines/vaccines, particularly during the COVID‐19 pandemic. Moreover, the current wave of medical research on COVID‐19 suggests the previous vaccine R&D ‘failures’ were driven by the modest demand for such vaccines and were not due to an inadequate IP‐incentive stream. The paper also suggests today's EU competition law rules on the horizontal exchange of information could be seen as an impediment to innovation and thus be temporary suspended.

## 1 INTRODUCTION

The coronavirus pandemic is a human tragedy of potentially biblical proportions, with Europe being one of its epicentres. It is indisputable that the world is behind in developing and being able to supply the diagnostics, vaccines, therapeutics, medical devices, and other suitably adapted medical supplies (medical technologies) needed to respond to the COVID‐19 pandemic currently sweeping across the globe.^1^

The development of vaccines targeting the COVID‐19 pandemic remains crucial.^2^ In an attempt to better address this unprecedented global human tragedy, Costa Rica, for example, has proposed the creation of a voluntary emergency Technology Intellectual Property Pool.^3^ Rutschman joins in this line of reasoning and advocates that less property‐like protection could effectively remove some of the most salient transactional obstacles to the development and commercialisation of new and better COVID‐19 vaccines.^4^ Moreover, while examining the market dynamics related to infectious disease products Darow, Sinha and Kesselheim argue that the ‘legislative initiatives launched over the past 15 years to overcome the shortcomings of the patent system have had limited success, in part because they do not adequately address the reasons underlying the disconnect between patents and the antimicrobial market’.^5^ Johnson and Bailey contend the current US patent law acts to limit the free flow of scientific research findings, and suggest a government‐funded reward system as an adjunct to the patent system to incentivise pandemic‐relevant research and its rapid publication.^6^ Rimmer points to the constant external and internal pressure on drug companies to view the coronavirus pandemic as a profit‐making opportunity.^7^ Further, a group of over 140 other organisations and individuals established an initiative calling on the WIPO to ensure that intellectual property (IP) regimes support, namely do not impede, efforts to both fight new coronavirus outbreaks and their consequences.^8^

However, the recent quickly assembled mosaic of coronavirus vaccine development projects^9^ might indicate the propulsive effects of global health crises on vaccine R&D and also shed light on the real systemic shortcomings that affect the development of vaccine targeting emerging pathogens. Despite the view described above that the IP law system^10^ of today is impeding COVID‐19 vaccine development, we may in fact observe a worldwide‐vaccine‐development race and an explosion of research activity. In contrast to all the above‐mentioned suggestions, scientists around the globe have actually been racing to develop vaccines against the novel coronavirus since its emergence in early 2020.^11^ This is a remarkably expedited timeline compared to the previous multiyear timeline typically needed to fully develop and produce a vaccine for testing.^12^ Does this then mean the IP law system in place today is not such an overwhelming obstacle to vaccine development and pharmaceutical innovation?

This paper joins in this critical debate and attempts to show that the intertwined static and dynamic efficiency and the ex ante versus ex post optimal innovation incentive stream may provide additional, insightful guidance for structuring the current discussion on IP rights and the COVID‐19 pandemic.

Our work contributes to the IP literature by addressing five key research questions: (a) Will COVID‐19 break the historical pattern of failing to produce a vaccine during the time of the outbreak of an infectious disease due to the lack of incentives; (b) can in fact the present international IP system be regarded as an impediment to innovation and thus must be altered to hasten the development of COVID‐19 vaccine (i.e., do we really need to overhaul the IP patent law); (c) is it more reasonable to change IP patent law in the middle of an outbreak under public pressure or to do so in ‘peaceful’ times; and (d) how to establish long‐term incentives to develop vaccines?

In this article, the analysis is as positive as it is normative. The interdisciplinary methodology^13^ employed can enrich a theoretical and comparative study of this kind by helping to draft better rules in day‐to‐day law‐policymaking. However, several caveats are appropriate. First, although scientific knowledge of COVID‐19 is growing, it is still incomplete at the time of publication of this paper. Second, the situation relating to COVID‐19 vaccine development is unprecedented in human history and hence any inferences derived from COVID‐19 may not be accurately extrapolated on future vaccine development in nonpandemic times. Third, pharmaceutical firms may instead on patent protection rely on trade secrets and confidential know‐how to protect COVID‐19 vaccines and their production.^14^ Fourth, potential infringements of for example messenger RNA (mRNA) vaccines may not be very likely to occur.^15^ Fifth, enforcement of patent rights by pharmaceutical firms may due to various strategic reasons not be very likely. Accordingly, this paper is therefore unable to conclusively argue about the appropriate patent law regime but seeks to offer the foundations for the ongoing debate.

This paper is organised as follows. In Section 2, we offer a general conceptual framework and discuss the recent literature on funding pools, patent pools and the COVID‐19 pandemic. Section 3 examines the roles of recent public policy, the compulsory licence, march‐in rights, European intellectual property, and Trade‐Related Aspects of Intellectual Property Rights (TRIPS)‐related case law trends concerning international pharmaceutical firms. This part focuses on the distortion of such firms' incentives to innovate and the fragile balance with the incentives for diffusion. This section also provides several, economically inspired, instrumental insights and a set of recommendations for an improved EU‐wide, supra‐national intervention. Section 4 concludes.

## 2 GENERAL CONCEPTUAL FRAMEWORK

In legal and economic terms, patent protection is an extremely powerful, sophisticated mechanism for providing incentives, motives for creating new ideas, products, inventions, designs and designs. Analytically speaking, a patent is a monopoly, a grant of exclusive rights in rem over intellectual creations, technical solutions and inventions.^16^ According to Douglas North (Nobel Prize winner for Economics), the establishment of these rights (patents) is also one of the most important foundations and reasons for Western civilisation's unprecedented success and prosperity.^17^

Moreover, the adoption of the ‘Statute of Monopolies of 1623’ (1623 c. 3, Regnal. 21 Ja 1) in Britain in 1623^18^ is considered one of the key enablers of the Industrial Revolution and hence the unheard‐of economic growth, a real explosion of economic activity and the continued increase in social well‐being.^19^ The granting of an exclusive right in rem (monopoly) enables the creator of an idea to enjoy a large part of its social value.^20^ This right in rem (assuming the strict and objective exercise of such rights) and the ensuing certainty that, if its technical invention is accepted by the market and economically viable, the inventor will be able to recover not only his initial ‘relation‐specific’ development costs, and the costs of manufacturing the product or invention, but also that they will be able to reap the benefits (if any) that the product/invention will bring—this ex ante opportunity for cost recovery and participation in potential profits are outstanding motivational mechanisms that act as incentives to potential inventors for their productive behaviour and innovation (which in the long run all increase economic activity, economic growth, and social well‐being).

The granting of patent protection is, analytically speaking, through the grant of a title to a particular invention, in fact the grant of a monopoly over it.^21^ Yet, since according to economic science every monopoly is theoretically and empirically (of course, except for a natural monopoly): extremely harmful, dangerous, a source of inefficiency, destructive of economic and economic activity, inhibitive/inhibits innovation, facilitates the appropriation of unjustified monopoly rents, enables moral hazard, opportunism and nepotism, and thereby directly reduces social well‐being,^22^ IP law must strike a balance between fostering innovation and the dissemination of ideas. This trade‐off between providing incentives to innovate and preventing monopolies is also the main reason for the strictly limited time of patent protection (up to 20 years) and for the evident rise in patent protection maintenance costs.^23^

Legal and economic analysis therefore enables us to understand the analytical reasons for granting these (otherwise economically damaging) time‐limited monopolies since providing incentives for innovative and productive creation is so important that it also outweighs (for a short period of up to 20 years) the negative impacts of such a monopoly (monopoly annuity, reduced use, and dissemination of such knowledge, possible opportunism and appropriation of unjustified annuities etc.). Monopolists thus enjoy annuities, profits in excess of a normal return on investment, while the monopolies so granted cause social costs by producing too few monopolised goods at an excessive cost.^24^ It also follows that the granting of patents—monopolies for ‘inventions’, which are not true inventions, but merely imitations of real inventions—is legally and economically totally unacceptable. The granting of patents (monopolies) for such imitations is a direct source of inefficiency, moral hazard and opportunism, transaction costs, the adverse selection problem, and in fact allows the rent‐seeking behaviour to remain unjustified. In these cases, this amounts to a complete redistribution, redistributive behaviour (and not the desirable productive behaviour, like with ‘real’ patents, which are genuinely new technological, innovative, and industrially applicable inventions that enhance social well‐being) that directly reduces economic activity and social well‐being. The granting of national patents, which are merely a copy, an imitation of some other foreign technical invention (thereby creating small national monopolies for which all incentives for creative, productive behaviour and investment are eliminated), is thus extremely damaging and, in the long run, devastating for the economy and the welfare of a given nation.

However, behavioural law and economics indeed suggest that creators do not consistently behave in the way traditional law and economics analysis assumes. Instead of rationally weighing up the objective costs and benefits of different courses of action, creators have instead been influenced by decision‐making heuristics and individual preferences that often led to suboptimal and inefficient creative behaviour.^25^ Moreover, Bechtold et al. show that authors and inventors chose to borrow when innovating was the optimal strategy, and chose to innovate even more when borrowing was the optimal strategy.^26^ They find that subjects are only mildly responsive to external incentives and that their choices between innovation and borrowing much more strongly correlated with their internal, subjective beliefs about the difficulty of innovating.^27^

### 2.1 Literature overview: COVID‐19 and IP law

The issue of the regulatory design of the current IP regimes and the potential impact of such regimes in times of a global pandemic had up until recently (the SARS outbreak in 2002) escaped the attention of law and economics scholarship. As Santos Rutschman argues, this lack of scholarly attention facilitated suboptimal decision‐making and significant sources of inefficiency.^28^ She argues that the world is ill‐prepared for the next disease outbreak when it comes to vaccines and IP laws. She also shows that one of the biggest problems is the lack of incentives for companies to develop a drug or vaccine.^29^ Santos Rutschman adds that we should invest more in finding a vaccine or other treatment to cure such diseases in peaceful times, when we know about a disease that still has small numbers, not when an outbreak occurs.^30^ Further, Santos Rutschman discusses the role and functions of The Coalition for Epidemic Preparedness Innovation (the CEPI).^31^

Halabi, Rourke, and Katz consider the 2012 Mers‐CoV and 2014–2015 Ebola outbreaks^32^ and identify several shortcomings of the actual policy responses. They suggest considerable information (asymmetric information problems) was not shared due to pitfalls in the patent law during times like this.^33^ They proposed solutions, such as funding and rewarding the collection of any useful data, establishing an internationally trusted database to report the findings to, and expanding the number of researchers while not discriminating against small or individual researchers.^34^

After the Ebola outbreak, the literature addresses the problems of new sanctions like compulsory licensing, its pitfalls, and other alternative solutions, but another important topic assumes a big role in battling a pandemic: free speech.^35^ Johnson and Bailey identify how (besides patent law and IP law) free speech is important for sharing the right information with the public to stop the spread of false news and related panic among the public.^36^ They argue that patent law actually slows down the development of a drug or vaccine because even if one lab group shares only a small amount of (unpatentable) information that helps others to use it and develop a vaccine, that will become patented and bring money exclusively to them.^37^ This then slows down the development of a new vaccine or drugs to fight the coronavirus.^38^ Namely, as Bailey and Johnston state, patenting a vaccine is economically speaking like holding a monopoly over it and therefore acts as a good motivation for developing it.^39^

Yet, when looking at the bigger picture, another problem regarding a vaccine for COVID‐19 might be identified, which makes the patent and IP issues look minor. After a vaccine is found/developed, it must still go through long medical trials, which takes months. Namely, even if a vaccine is developed today, it can take 1 year or so to reach the market and be available for people to use. The result is that, even if the patent law does slow the finding of the right vaccine down by 1 or 2 month, this will not really cause much more damage because in about 1 year (the approximate duration of drug trials) there will not be many critical cases and not much by way of additional damage if the vaccine comes onto the market an additional 1 month later. Yet, in critical times such as the present coronavirus outbreak, it is crucial to encourage the development and production of vaccines. Still, it is not an easy road to take since there are many pitfalls and holdbacks in patent markets, especially when it comes to products for infectious diseases. Darrow, Sinha, and Kesselheim discuss the issue of the downfall of the infectious disease drug/vaccine markets,^40^ that have often proven to be unpredictable and under‐resourced. This uncertainty scares developers, which explains why there are very few vaccines for very dangerous and fatal diseases, such as tropical diseases, regarding which most patients are unable to pay for treatment, making the market for the vaccine under‐resourced and unattractive.^41^ Further, Magnusson argues that law plays a vital role in inducing the production of vaccines and provides an overview of legislative tools designed to incentivise vaccine developers.^42^

Beldiman advocates the establishment of patent pools as the most effective method for tackling the problem of infectious diseases.^43^ Beldiman also discusses different economic viewpoints, their (anti‐)competitiveness, how they are enforced, and describes many failed proposals to the WHO.^44^ She also questions whether virus samples, even if of natural origin, should be patented.^45^

Tietze et al. perform an ad‐hoc patent analysis suggesting that the majority of coronavirus‐related patents in the field concern organic chemistry, and the development of methodologies and drugs for the prevention, diagnosis, and treatment of viruses.^46^ They also identify a time‐lag between the outbreak and the materialisation of patent applications, which is consistent with the processes of the UK Patent Office.^47^ The large number of references to nonpatent literature published after outbreaks may, according to Tietze et al., be interpreted as indicating the urgent need for scientists to put information into the public domain and make it accessible quickly to a wider audience.^48^ On the other hand, Xue and Ouollette argue that a large number of ‘missing’ vaccines is likely due to more than a lack of scientific opportunities.^49^ They detect two key aspects of vaccines that help account for their ‘anaemic’ development pipeline: (1) they are preventatives rather than treatments; and (2) they are generally durable goods with long‐term effects rather than products purchased repeatedly. They suggest that both aspects make vaccines less profitable than repeat‐purchase treatments, even when given comparable IP protection.^50^ Whereas, Santos Rutschman suggests that analysis of the earlier Ebola and Zika outbreaks shows that developers expect a decrease in funding for research for a cure once the number of infected starts to decline (the components needed to develop a new vaccine are also patented and thus expensive).^51^

Finally, Walsh et al. suggest that existing IPR frameworks represent access barrier to IPRs during public health crisis and argue that a systemic re‐evaluation is required where positive and equitable legal measures protective of the public(s) interest(s) should be built within IPR frameworks that also address non‐IPR barriers.^52^ McMahon contemplates that current patent law arguably gives considerable unfettered discretion over how an invention is used to patent holders despite the significant potential health and ethical implications arising.^53^ Also Correa argues that the current IPRs system does promote research into more effective and efﬁcient treatments and calls for alternative mechanisms that should be established (such as ‘open‐access’ models) to encourage more R&D in diseases disproportionately affecting developing countries.^54^ Light proposes nonprofit health care and pharmaceutical development that could ameliorate current health disparities and which would employ the entrepreneurial collaboration for public health markets and will invert IP to public health IP to maximise health gain instead of profits.^55^

### 2.2 IPRs and the anticommons

The conventional tragedy‐of‐the‐commons problem emerges when more than a single person or agent is assigned usage rights.^56^ In these circumstances, there is a tendency for commonly owned assets to be overused, even to the point of destroying their value.^57^ Examples are familiar: medieval common pasture, fishing grounds, oil pools, aquifers, hunting territories, and locational amenities.^58^ For a century, economists have been ready to offer solutions to this tragedy. The value shortfall emerges due to the absence of effective management of the resource; use must be limited. One approach to management that will ensure efficiency is to assign ownership rights.

However, the literature also shows the flip side of the tragedy of the commons. One can also find situations where the existence of numerous interested parties with control rights (property rights) makes it very costly (transaction costs) to access an asset, with the result that the asset is underused.^59^ This instance, where many individuals have to agree before an economic resource can be employed in a new way, is labelled the ‘tragedy of the anti‐commons’.^60^ For example, as noted, the patent system establishes property rights in inventions and should thereby provide strong incentives to investors to find valuable new products or processes. Yet, the establishing of such extensive property rights for technical inventions might lead to a ‘tragedy of the anticommons’. Current inventors, as Leitzel points out, as for example in vaccine research, build upon the stream of previous inventors/inventions and, if there are multiple patent holders whose own patented products are required components for a new invention, the tragedy of the ‘anticommons’ potentially arises.^61^ Inventors might then be driven away from areas where patents already exist due to the high transaction costs and possible hold‐out demands made by numerous patent holders.^62^ In addition, Heller and Eisenberg, for example, argue that granting too many patent rights in premarket or upstream biomedical research might stifle the discovery of life‐saving products downstream.^63^ Hence, strong and protracted patent rights in the pharmaceutical industry (and in vaccine development) might not necessarily lead to robust incentives for innovations. Still, in her historical empirical study, Moser shows that inventors are most likely to use patents in industries where innovations are ‘easy to reverse‐engineer and secrecy is ineffective relative to patents’.^64^ As she suggests, ‘in the late 19th‐century, scientific breakthroughs, including the publication of the periodic table, lowered the effectiveness of secrecy in the chemical industry’.^65^ Difference‐in‐differences regressions suggest that this change resulted in a significant shift towards patenting.^66^

Yet, in an experimental study Vanneste, Van Hiel, Parisi and Depoorter show that ‘anticommons situations generate greater opportunistic behaviour than an equivalent commons dilemma, and anti‐commons dilemmas yield a greater risk for underuse compared to commons dilemmas’.^67^ In other words, their behavioural and empirical study shows that the ‘tragedy of the anti‐commons presents a greater social threat (underuse from blocking the use of resources by posting very high selling prices) than the commons dilemma (overuse of resources).^68^ They also argue that the anticommons might be considered as having even more serious and problematic consequences than the commons dilemma.^69^

## 3 TOWARDS THE OPTIMAL REGULATORY FRAMEWORK

This section offers a set of recommendations for an improved public policy framework and for informed political discussion. It discusses vaccine production failures and incentive mechanisms and investigates whether COVID‐19 will be able to break the historical pattern of failing to produce a vaccine during the time of the outbreak of an infectious disease due to the lack of incentives.

### 3.1 The TRIPS agreement and the pharmaceutical industry

Governments should act swiftly to put legislation and plans in place to provide a long‐term incentive stream for infectious diseases and ensure that patents do not become barriers to access to such products. Namely, to balance investors' rights with public law, the question of the extent of IPRs protection should be answered. There is a general theory of IPRss which suggests countries on different levels of economic development have varying best interests with respect to the strength of IP protection, and that these best interests change over time.^70^ A description of the current European IP law on patents lies beyond the contemplations of this paper and can be found elsewhere;^71^ still, it must be recognised that the grant of patents in medical technology and biotechnology have posed ‘significant challenges for the European patent system’.^72^ Namely, the European Patent Convention's (hereinafter EPC)^73^ grant of patents for pharmaceuticals and medicines brought patentees into conflict with end‐users either by de facto estopping access to medicines or charging excessive and unaffordable prices.^74^ The literature also identifies industry‐specific patenting strategies whereby firms apply for several patents in relation to a single medicine.^75^ In this respect, the EU Commission suggests that such patenting patterns are usual in the pharmaceutical sector and engaged in so as to block or delay the market entry of generic medicines, creating cost‐ and wealth‐decreasing implications for the general public.^76^ Disappointingly, the EU Commission merely suggested that practices in the pharmaceutical sector be monitored and an EU patent‐litigation system be introduced that would cut the costs and be more efficient for citizens.^77^

Regarding international legal institutions, one should note that the Patent Cooperation Treaty (PCT)^78^ and the Agreement on TRIPS^79^ introduced ‘compulsory licences’^80^ as a remedy for any restrictions on life‐saving medicines.

More specifically, Article 31 of TRIPS (Other Use Without Authorization of the Right Holder) among others provides that the person or company applying for a licence must have tried, within a reasonable period of time, to negotiate a voluntary licence with the patent holder on reasonable commercial terms.^81^ Only if that fails can a compulsory licence be issued and, even when a compulsory licence has been issued, the patent owner is entitled to dully compensation; the TRIPS Agreement provides ‘the right holder shall be paid adequate remuneration in the circumstances of each case, taking into account the economic value of the authorization’, but it does not define ‘adequate remuneration’ or ‘economic value’.^82^ Moreover, compulsory licensing must meet certain additional requirements: the licence's scope and duration must be limited to the purpose for which it was granted, it cannot be given exclusively to licensees (e.g., the patent‐holder can continue to produce) and it should be subject to legal review.^83^

One problem is that the TRIPS Agreement does not specifically list the reasons that might be used to justify compulsory licensing.^84^ However, Article 31(f) of TRIPS, which states that medicines produced under a compulsory licence must chiefly be for the domestic market, initially created problems for countries unable to manufacture a patented medicine themselves.^85^ Yet, since 2003, when the WTO member states agreed that countries devoid of domestic capacities to manufacture drugs should be able to import cheaper generic drugs made under compulsory licences in other countries,^86^ a system of compulsory licences is de facto effective that also allows countries to manufacture patented pharmaceutical products under compulsory licences for export to developing countries.^87^ As Bently and Sherman stress, the compulsory licensing regime now covers ‘patented products and products made using patented processes in the pharmaceutical sector, including active ingredients and diagnostic skills’.^88^

### 3.2 Compulsory licence and march‐in rights

The essence of patents rights is to exclude others from making, using or selling a patented invention, except with the patent holder's authorisation in carefully negotiated licensing agreements to ensure proper compensation for the efforts and costs invested in developing the patented invention.^89^ However, as already shown, the TRIPS Agreement enables governments to forcibly license (via the institution of ‘compulsory licences’) a patented invention in times of need, particularly during a threat to public safety. The question is: Should governments during the COVID‐19 pandemic resort to the use of these available, albeit rarely used, compulsory licensing provisions?

In addition, US laws grant the US government ‘march‐in rights’.^90^ March‐in rights is a provision of the Bayh‐Dole Act of 1980 and codified in 35 U.S.C. §203.^91^ March‐in rights give the US federal government the right to grant patent licences to other parties or take licences for themselves if the patented invention was researched and developed with the help of federal funding. Yet, from a dynamic efficiency perspective, such a ‘march‐in‐confiscation’ of patents might remove the incentives to innovate and thereby damage the innovation ecosystem that has generated breakthrough therapies and enabled scientists to work so quickly on COVID‐19.^92^ It is quite telling that no US Administration has ever actually employed the Bayh‐Dole march‐in rights for this purpose.^93^ Namely, as the traditional law and economics literature suggests,^94^ in such a scenario (enforcement of the march‐in option) incentives to innovate, might be distorted and the enforcement of such march‐in rights might be viewed as a source of dynamic inefficiencies, increased transaction costs and greater uncertainty (for innovators and research activity). In other words, those opposed to march‐in rights fear that such a policy may reduce long‐term access to critical innovations by weakening incentives to invent and transferring new technologies abroad.^95^ Kitch, for example, suggests that any form of march‐in rights destroys the prospect function because the patent owner then loses their ability to control who can use their patent.^96^ One may envisage that after licensing university patents, private firms spend enormous sums on additional research and development and, as Reinhart suggests, if the government were able to arbitrarily march‐in and seize IP due to the potential march‐in (exercise of the compulsory licensing option), the ensuing greater uncertainty would mean they would ‘hesitate to fund the research that has brought us so many innovations’.^97^ That would also mean ‘far fewer partnerships and licensing deals between companies and universities’.^98^ As to empirical findings on compulsory licensing, it is noted that the results are ambiguous because compulsory licensing may either encourage innovation by increasing competition or discourage innovation by reducing the expected returns on R&D.^99^

On the other hand, Bond and Sagi suggest the effects of compulsory licensing on global welfare are not always beneficial: while a relatively lax compulsory licensing policy increases world welfare, a compulsory licensing policy that is too strict can lower it by inducing the suboptimal switch from licensing to entry.^100^ In her empirical study, Chien finds no uniform decline in innovation by companies affected by compulsory licences and very little evidence of a negative impact on their innovation activity.^101^ Still, Auth for example, argues that, in the face of a global pandemic, ‘health or safety needs’ may indeed provide strong political pressure for the exercise of march‐in rights and grant of a compulsory licence if more patent owners, like Gilead, take a protectionist patent stance.^102^ Nevertheless, the availability of this measure may induce companies to voluntary (albeit strategically) suspend their patent rights during this global public health emergency to avoid government marching in.^103^

### 3.3 Strategically and temporarily waiving patent rights

As suggested, governments can employ the compulsory licensing mechanism (and march‐in rights) and use any patented IPR, and for that they only must pay a reasonable royalty for its use. But some IP holders have decided to temporarily suspend enforcement of their IP rights. That is, some patent owners are responding to the current COVID‐19 pandemic in a surprisingly benevolent way, essentially making government's exercise of compulsory licensing and march‐in rights options unnecessary. Namely, seeking injunctive relief and stalling COVID‐19 vaccine production may have serious detrimental effects on firms reputation and public relationship. Moreover, the relevance of such litigation might be dubious, since it well may be that by the time the litigation reaches trial, the patent may not be worth much to fight over due to mutant variants which are able to evade the ambit of the vaccine protected under the patent. Furthermore, enforcing patent to recoup R&D costs may not be that necessary under circumstances where there is a significant external funding and demand for the COVID‐19 vaccine readily developed. For instance, Auth reports that the drug manufacturer AbbVie has taken a ‘bold public health stance by suspending enforcement of its global patent rights on all formulations of the HIV medication, Kaletra (Aluvia) while the drug is being evaluated as a candidate to treat COVID‐19 in several clinical trials’.^104^

One may wonder whether such decisions amount to a strategic temporary waiver of enforcing IPRs given that the prospect of the IP being violated by the government may appear to bring extra risks to the IP holder.^105^ Yet, since compulsory licenses may be taken out by a particular state if the patent holder for example, plans to enforce its patent, such option of invoking compulsory license renders firm's de facto patent enforcement strategically unreasonable and unviable. Moreover, one may, as Auth suggests, wonder whether AbbVie's decision to suspend its patent rights over Kaletra is an act of ‘pure benevolence, mounting public pressures, or because at least some of their clinical trials suggests that Kaletra may not be effective in treating COVID‐19?’.^106^

Further, in the current global pandemic due to pure altruism or very strategic reasons firms could actually show society their social responsibility by suspending their IPRs. In addition, firms may refrain from filing a patent for a successful vaccine and/or manufacturing process to avoid having to disclose, in precise terms, how the respective invention works.^107^ Thus, due to unique circumstances posed by the COVID‐19, the motivations behind a patent holder to enforce its patent might not be as significant as compared to more orthodox circumstances.

### 3.4 The COVID‐19 pandemic and legal change

Is it socially advantageous to change the current IP patent system in light of the altered circumstances (the COVID‐19 pandemic)? While answering this basic question, Shavell relies on a simple argument regarding past compliance.^108^ As Shavell suggests, ‘past compliance with legal rules tends to reduce the social advantages of legal change’.^109^ The general implications are that legal rules should be more stable than would be appropriate were the relevance of past behaviour not recognised, and that a policy of grandfathering, that is, of permitting noncompliance, should sometimes be employed.^110^ Cafaggi, Nicita and Pagano show that uncertainty plays a crucial role in the optimal timing of law‐making.^111^ This is because investing in a new legal rule implies sunk costs and the switch to a new legal regime has nonzero opportunity costs. Evidently, these costs cannot be recovered if the law later proves undesirable or is subsequently repealed.^112^ As already stated, the process of law‐making is analogous to investment.^113^ More specifically, law‐making shares three critical characteristics with investments in physical assets: irreversibility of the investment (sunk costs); uncertainty about future returns; and discretion with respect to the timing of the investment. Once enacted, the costs sunk into certain legislation cannot be recovered if it turns out the legislation is ineffective or worse.^114^ Thus, the change in any legal rule should be 'slower than would be indicated by a simple discounted, net present value’.^115^ Further, the notorious transaction costs and asymmetric information problem are exacerbated in times of uncertainty (the COVID‐19 pandemic‐panic) and makes such hasty changes even more counterproductive.

Hence, making hasty changes to the current IP law regime (e.g., proposed annulment or complete suspension of patents for COVID‐19 vaccines) during pandemic and under current severe information asymmetries might prove to be counterproductive and distortive. Parisi, Fon and Ghei convincingly show that the greater the uncertainty, the greater the cost of giving up the option of waiting; and the greater the expected value of the law over time, the greater is the value of waiting.^116^ Of course, one may regard the COVID‐19 pandemic as a necessary stressor needed to achieve the resilience and antifragility of IP law. Yet such resilience triggered by the COVID‐19 stressor will be achieved ex post when the information needed for potential change is readily available. In addition, as we have shown, current TRIPS Agreement's provisions on compulsory licensing may already provide effective and efficient tool to reduce the shortcomings of traditional exclusionary patent regimes. Namely, compulsory licensing is an effective remedy for any restrictions on life‐saving vaccines and will also not result in a uniform decline in innovation by companies affected by compulsory licences and has generally also as a minor negative impact on their innovation activity. However, as shown the employment of march‐in‐rights should be due to their structural shortcomings and potential distortions of innovative activity, avoided.^117^

In addition, the current vaccination race, in which over 200 firms^118^ have been competing to find a vaccine, may be regarded as circumstantial evidence that the present IP regime should not be regarded as the sole obstacle to vaccination‐innovation.^119^ The fact the current rate of medical research on COVID‐19 is considerably faster than in any previous epidemic might imply that earlier vaccine R&D ‘failures’ in epidemics like Ebola, Zika and MERS were driven by the modest demand for such vaccines (and did not entail the problem of an inadequate IP‐incentive stream)—the demand side, not the supply side of the market problem. Due to their high mortality levels, prior epidemics like Ebola, Zika, MERS and H1N1 infected a very small number of people^120^ and were also successfully contained before becoming a global pandemic. In these circumstances, low‐cost containment measures appeared to be more effective than extensive R&D vaccine expenditures.^121^ This means that the suboptimal rate of vaccine R&D investments noted in the Ebola, Zika and H1N1 outbreaks might, as otherwise often argued by some scholars, not be caused by the overly exclusive property‐like protection nor is the patent system incapable on its own of inducing the R&D of vaccines that societies collectively need,^122^ but might be a materialisation of the demand side of the market problems and fast containment (combined with the very small number of infected people).^123^ The importance of market size on the HEN innovation rate can thus hardly be overstated.^124^ Insightfully, Acemoglu and Linn for example while providing a measure for radical innovations find significant and relatively robust effects of market size on pharmaceutical innovation.^125^ They show that a ‘1 percent increase in the size of the potential market for a drug category leads to a 6 percent increase in the total number of non‐generic drugs in that category’ and that ‘a 1 percent increase in the potential market size is associated with a 4–6 percent increase in the entry of new molecular entities (radical innovations)’.^126^ Moreover, the rate of development and commercialisation of inventions in the pharmaceutical industry might decline substantially in the absence of the patent system. For example, Mansfield in his empirical study shows that patent protection was essential for the development of the lion's share of inventions in the pharmaceutical industry.^127^

Finally, it is clear that the current race for a COVID‐19 vaccine, involving more than 200 pharmaceutical firms in competition, should be welcomed since, as a genuine antidote for market failures, competition assures low prices, availability and high quality.

Thus, the question is not whether one really needs to change the current IP law but whether there is a particular need for case‐specific regulations and policy interventions/recommendations targeted at bringing COVID‐19 vaccine to the market faster.

First, obstacles to the development and commercialisation (and consequent public availability) of vaccines might not be caused by the shortcomings of the current IP law but might instead be caused by potential weaknesses or imperfections of regulatory agencies. Namely, policymakers and vaccine‐approving authorities might be prone to ‘type I and type II errors’.^128^ For example, a vaccine‐approving authority can make two types of error: (a) type I error—approving vaccines that are too dangerous to be put in the market (sanction negligence criterion); or (b) type II error—not approving drugs that should be allowed (sanction gross negligence criterion). Consequently, a vaccine‐approving authority might play it on the safe side and approve too little (type I error) and too late/too much (type II error). In the United States, the FDA for example, might be only liable if it did too little and is hence inclined to require more examinations of the COVID‐19 vaccine trends than is optimal.^129^ Literature suggest that, for example, the drug approval system might put enormous stress on Type I errors and largely ignores Type II errors, thereby raising the cost of drug testing and delaying the availability of safe and effective drugs.^130^ Hence, a more balanced set of regulatory vaccine approval standards, accounting for the consequences of both Type I and Type II errors,^131^ could result in reducing high costs and long delays of introducing new vaccines and could also lower the costs of clinical testing.^132^ Moreover, regulatory agencies (e.g., FDA) also award data exclusivities that are independent of IPRs but may have a similar proprietary flavour. Namely, during the period of exclusivity the FDA may not rely on an innovator's safety and efficacy data to approve a competitor's product. Although, such data exclusivity is designed to enable innovators to recoup the investments they made into developing new products and testing product safety and efficacy, competitors must conduct their own safety and efficacy research and testing to obtain FDA approval and, obviously, not infringe the patents owned by the innovator. Such data exclusivity might then exacerbate regulatory agencies' account of both Type I and Type II errors and might consequently slow down the development and commercialisation of vaccines.

Second, substantial social costs arising from COVID‐19 might have been avoided with proper ex ante investments in infectious disease basic/fundamental research (a traditional public good) if earlier coronavirus vaccine research had not been shut down due to a lack of funding.^133^

Third, the immediate institutional responses have been quite revealing and, if implemented, could lead to serious distortions. For example, Germany and Israel proposed suspending all patent rights, Canada permitted the use of patents to the extent required by the health emergency, and the European Union found a resolution in patent pooling, which is the voluntary sharing of IP to support the faster manufacture of potential COVID‐19 vaccines.^134^ Another potential solution, as already noted for the identified market failures is, apart from competition and patent pools, compulsory licensing which, when taking the extremely high vaccination development costs into account, retains the classic property‐rights‐driven incentive stream.^135^ Yet, one could argue that compulsory licensing might have its own ‘strategically’ driven shortcomings.^136^

Fourth, another possible institutional arrangement is to facilitate crisis‐critical patent pools which can then be made available to interested stakeholders which wish to employ that IP.^137^

Fifth, targeted ex ante research subsidies, public‐private partnerships, investment in basic/fundamental research should be provided as a public good (e.g., fundamental research at universities financed and provided as a public good) and address the pathogens regarding which the demand side of the market failure (e.g., SARS and MERS) whereby adequate incentives cannot be provided for private R&D investment. To generate long‐term incentives, such fundamental research should be consistently funded throughout the whole process of vaccine development.

Sixth, the employment of behavioural (nudging) instruments (reputation among peers, legacy, social appreciation, honours) to boost such basic‐fundamental research—public research grants, inventors awards (e.g., the EPO's European Inventor Award—Inventors against Coronavirus), public‐innovation hubs, ‘wild cards’, and scholarships. However, such ‘inventor awards’ or best entry tournament proposals (which effectively give an advance payment or purchase commitment), may create a winner‐takes‐all framework. Such a winner‐takes‐all framework does not induce competition for subsequent improvements and such ‘all‐pay‐auctions’ might deter industry from participating and thus provide suboptimal incentives to innovate. The employment of wild cards might bring adverse effects. Namely, wild cards or ‘transferable patent extensions’ place the 'cost of developing products on third‐party payers who are buying the existing products whose patent is extended’ and deters competition with respect to later improvements.^138^

### 3.5 Adjusting the long‐term incentives

The costly and complicated development of the infectious disease vaccine market has traditionally meant it is seen as one of the least profitable pharmaceutical markets. If one divides vaccine development into three stages, the first being antigen discovery, formulation and animal studies, the second being early development, in which optimisation, the first and second phases of development take place with the third being late development, entailing final manufacturing, licensing and third phase of development take place, there are very different financing structures in each stage. In the first two phases, the financing is well covered by investors such as CEPI, the Bill&Melinda Gates Foundation, PATHS and others, but in the third stage of late development, which actually accounts for about 70% of total development costs, there is a big gap in financing as there is no too little funding for late‐stage trials.^139^ This results in the dropping out of smaller participants who do not possess adequate finance for vaccine development and commercialisation. Therefore, one suggestion to improve long‐term incentives is to ensure consistent funding during the whole process of vaccine development.^140^

Moreover, it should be recalled that a vaccine is not a treatment for the already sick but is medicine that is preventive, a collateral effect of which is herd immunity—the vaccination will not only benefit the vaccinated individual, but also the people around them as they will not be a possible spreader of the disease (a positive externality and a solution for potential free‐riding problem).^141^

Another characteristic of a vaccine is its one‐off, long‐term effect—people might only have to be vaccinated once and their immunity will be long‐term compared to most medication, which must be taken multiple times, continuously and will cure only one person and not create the collateral immunity of anyone else.^142^ This directly disincentivises pharmaceutical firms from R&D expenditures in the infectious disease vaccine market. To provide incentives, Xue and Ouellette suggest that innovators should be compensated according to the social surplus their inventions generate, whereas consumers should have access to subsidised or low‐price vaccinations.^143^

However, as already stated, the current fast rate of medical research on COVID‐19, which considerably exceeds that seen in any previous epidemic, might mean that previous vaccine R&D ‘failures’ in the Ebola, Zika, MERS and SARS epidemics were driven by the modest demand for such vaccines (and thus not due to an inadequate IP‐incentive stream) and the high development costs^144^—the demand side, not the supply side of the market problem. It is insightful to learn that, as of today, in total there are 44 vaccines in clinical trials on humans and at least 92 preclinical vaccines are under active investigation on animals.^145^

At the time of writing vaccines in final phase‐3 trials are: Moderna, BioNTech in collaboration with Pfizer and Fosum Pharma with the vaccine BNT162b2, Johnson&Johnson, Novavax and AstraZeneca's one (ChAdOxl).^146^ Thus, this ‘vaccination race’, where more than 200 firms compete for finding the vaccine (11 vaccines in third stage trials) could be regarded as circumstantial evidence that the current IP regime actually provides a stable stream of incentives and should not be viewed as the sole obstacle to vaccination‐innovation.

Yet, when discussing long‐term incentives to innovate one also needs to address the ‘tragedy of the anti‐commons’. As Leitzel points out, current inventors, such as in vaccine research, build upon the stream of previous inventors/inventions and if there are multiple patent holders whose own patented products are necessary components for a new invention, a tragedy of the ‘anti‐commons’ potentially emerges. Inventors might then be driven away from those fields where patents already exist due to the high transaction costs and potential hold‐out demands made by numerous patent holders. The literature suggests that such a tragedy of the anticommons might be reduced with the employment of patent‐pools, compulsory licences, direct subsidies, reward and reputational mechanisms, and sanctions.

When considering all of the above, one may argue that policies which increase the value of markets for pharmaceuticals might in fact be employed as long‐term incentive mechanisms to induce vaccine‐related R&D. For example, Kremer et al. suggest that the ‘US Orphan Drug Act, which went into effect in 1983, actually created a number of financial incentives for pharmaceutical companies to develop drugs for rare diseases like Huntington's, ALS (Lou Gehrig's disease), and muscular dystrophy—diseases which affect fewer than 200,000 people in the United States and therefore have a limited market’.^147^ The primary attraction for companies is the promise of 7 years of market exclusivity and hence over ‘200 orphan drugs have been developed since 1983, while fewer than ten were introduced in the decade preceding passage of the act’.^148^ Kettler argues that biotech's in particular have responded to the incentives provided by the US Orphan Drug Act.^149^ Moreover, Kettler and Marjanovic note that as of 2000 biotechnology companies had sponsored 70% of the more than 900 orphan‐designated projects in the United States, and 50% of all approved biotechnology products held orphan status.^150^

Yet, a key concern from the perspective of improving public health is not just providing incentives for innovation but also linking incentives to access to products once they have been developed. Further, as Towse and Kettler suggest, the first generation of vaccines will not be ideal, and thus there should be a strong effort to give incentives for subsequent innovation.^151^ As we discuss further in the next section, advance purchase commitments may be particularly suitable for encouraging R&D on neglected pathogens.

### 3.6 Advance purchase commitments and targeted subsidies

Empirical studies show that the more severe the COVID‐19 pandemic is, the bigger is the share of new drug development coming from small firms.^152^ Bryan et al. also suggest that during the current COVID‐19 pandemic there have been too many quick‐to‐develop projects involving small firms.^153^ They argue that this inefficiency requires a change in governmental innovation policy and emphasise targeted subsidies and advance purchase commitments as mechanisms that should correct directional incentives. An advance purchase agreement is essentially a commitment to purchase a product that at the time of signing the contract does not yet exist.^154^ It is interesting that many advance purchase agreements have indeed been formed by the European Union and the United States and the most promising vaccine developers. For instance, on 14 August 2020 the EU reached such an agreement with the pharmaceutical company AstraZeneca while several others are in the bargaining process.^155^ Thus, the European Union funded the development, manufacturing and deployment of vaccines against COVID‐19 so as to accelerate and ensure their safety and effectiveness. The US government signed similar advance purchase agreements with BioNTech‐Pfizer, NovaVax, AstraZeneca and Sanofi‐GSK.^156^

Do such advance‐purchase agreements really provide incentives to innovate or might they actually deter innovation? Economically speaking, advance purchase agreements may reduce ex ante economic uncertainty and give investors' confidence in the returns they can expect if the relevant scientific challenges are overcome.^157^

Advance purchase agreements also allow biotech's, pharmaceutical firms, and emerging market suppliers to create whichever R&D structures they believe will be most effective.^158^ In addition, the existence of an advance purchase commitment implies there is a ‘*de facto* market for intermediate research outcomes, as a small company can sell promising leads in a neglected disease covered by an advance purchase commitment in the same way that a small company would sell leads in a major developed country therapeutic area such as cancer or heart disease’.^159^ This would mean that small, originally unfunded COVID‐19 vaccine developers would also be induced to innovate since they can sell promising research avenues (intermediate research outcomes) to the holder of such an advance purchase agreement.

Still, the exclusive purchase agreements might indeed distort the competition and be a source of inefficiency (crowding‐out effects and negative externalities). For example, the United States and the European Union literally monopolised the market by agreeing to be ‘the first in line’ when the vaccines become available. Such agreements might also distort SMEs' incentives to innovate and, by depriving other countries from purchasing vaccines, cause negative externalities. That means it is critical to avoid exclusive advance‐purchase agreements (which also raise competition law concerns—the abuse of a dominant position, refusal to deal etc.) and thus de facto monopolising the vaccine (resulting in high prices, exclusion of others' access to the vaccine, and dubious quality).

### 3.7 Temporary suspension of provisions on the horizontal exchange of information

The direct or indirect exchange of information between competitors under the EU's competition rules is one of the most controversial issues, raising one of the most challenging competition law questions.^160^ The most fundamental change here is the replacement of the centralised notification system for a ‘legal exemption system’.^161^ The horizontal effect of the EU's new legal exemption system upon the provision of stable and optimal incentives (dynamic efficiency) to innovate has largely been exempted from the current scholarly debate. Namely, the legal exemption system and associated threat of ex post punishments may introduce ex ante uncertainty and generate negative effects in terms of information production and innovation as concerns the COVID‐19 vaccine. The fact that firms can no longer apply for a negative clearance and must self‐assess the legality of their co‐operation introduces the risk that they might refrain from engaging in efficient forms of information exchange.^162^

Further, the introduced block exemptions to categories of R&D may indeed reduce the uncertainty for some firms, but not for all since legal uncertainty remains high as the existing market share threshold and the definition of the relevant market are difficult to determine ex ante. The existing case law on information sharing may also itself increase ex ante uncertainty and hence have a chilling effect on entrepreneurial activity because any information sharing might, in certain circumstances, infringe EU competition rules.^163^ Information which is not historical and relates to matters such as price, capacity and cost is commercially sensitive and therefore its exchange is more likely to infringe than other types of information. The exchange of individual data about particular undertakings is more problematic than aggregated data. Another relevant factor is the frequency of any information exchange. A survey of several landmark decisions shows that practically any type of information directly or indirectly capable of being seen as collusive behaviour cannot be exchanged without causing concerns for the EU's competition authorities.

Obviously, the application of such wide, all‐inclusive and vague criteria concerning when an exchange of information between undertakings may be regarded as an infringement of Article 101 TFEU may be a source of uncertainty and a needless rise in transaction costs.

The second source of uncertainty, as already stressed, results from adoption of the new ‘self‐assessment system’, which in reality has exacerbated the problem. This self‐assessment system may, as already noted, actually be the most problematic for horizontal entrepreneurial activity because firms face a high level of uncertainty.

To sum up, EU competition law provisions on information exchange and R&D horizontal agreements through the self‐assessment procedures and the vague evaluation criteria for when an information exchange amounts to an infringement of those EU rules may be perceived as one of the potential, thus far overlooked, impediments to developing vaccines. Thus, the current provisions concerning the exchange of information might be temporarily suspended during the COVID‐19 pandemic.

## 4 CONCLUSIONS

This paper suggests that today's patent law regime and the TRIPS Agreement are not an insurmountable obstacle to accessing a successful COVID‐19 vaccine. Via the existing compulsory licensing and the employment of patent‐pools, research subsidies, reward mechanisms and reputational sanctions, governments can take the steps needed to effectively overcome any IP barriers (such as market price deflation) to ensure access to crucial medicines/vaccines, especially during the COVID‐19 pandemic.

The publicly advocated change or even abolition of the current patent law regime under serious information asymmetries might prove to be counterproductive and distortive. Namely, the greater the uncertainty, the greater the cost of giving up the option of waiting; and the greater the expected value of the law over time, the greater the value of waiting. Of course, one may regard the COVID‐19 pandemic as a necessary stressor for achieving the resilience and antifragility of the IP law. Yet such resilience triggered by the COVID‐19 stressor will be achieved ex post when the information needed for any change is readily available. In addition, as we have shown, the existing legal institutions (e.g., compulsory licence, advance purchase agreements) may already provide effective and efficient tools to reduce the shortcomings of traditional exclusionary patent regimes.

The present vaccination race that has seen over 200 firms compete to find a vaccine may also be regarded as circumstantial evidence that today's IP regime should not be viewed as an obstacle to vaccination‐innovation. Namely, the current fast rate of medical research on COVID‐19, which considerably exceeds that seen in any previous epidemic, might mean that the earlier vaccine R&D ‘failures’ in the Ebola, Zika, SARS, MERS epidemics were driven by the modest demand for such vaccines (and were thus not due to an inadequate IP‐incentive stream)—the demand side, not the supply side of the market problem. The earlier epidemics infected a very small number of people and were also successfully contained before they became a global pandemic. In these circumstances, low‐cost containment measures appeared to be more effective than extensive R&D vaccine expenditures. Hence, the suboptimal level of vaccine R&D investment noted in the Ebola, Zika and H1N1 outbreaks might, as often argued in the literature, not be caused by overly exclusive property‐like protection, but might be a materialisation of the limited demand and fast containment.

In addition, the paper suggests the current EU competition law provisions on information exchange and R&D horizontal agreements through the self‐assessment procedures and vague evaluation criteria concerning when an information exchange amounts to an infringement of those EU rules might be perceived as a potential, thus far overlooked, impediment to developing vaccines. Finally, one should clearly welcome the current race for a COVID‐19 vaccine since, as an effective antidote for market failures, competition assures low prices, availability and high quality.

## Data Availability

Data sharing is not applicable to this article as no new data were created or analyzed in this study.

## ENDNOTES

1 On 11 March 2020, the World Health Organization declared a pandemic and, by the summer of 2020, the pathogen had spread across the globe, claiming the lives of over 1 million people; World Health Organization, Coronavirus Disease (COVID‐19): Situation Report‐168, 2020.

2 Peter F. Wright, ‘Vaccine Preparedness—Are we Ready for the Next Influenza Pandemic?’ (2008) 358 NEJM 2540.

3 Formation of the TIPP would in the first instance be coordinated by the WHO, after which operational implementation might be assigned to other coordinating entities; James, Love, *President and Minister of Health of Costa Rica ask WHO to Create Global Pool for Rights to COVID‐19 Related Technologies* (KEI 2020) <https://www.keionline.org/32556>

4 Ana Santos Rutschman, ‘Property and Intellectual Property in Vaccine Markets’ (2020) TA&MUJPL.

5 Darow J. Jonathan, Sinha S. Michael, and Aaron S. Kesselheim, ‘When Markets Fail: Patents and Infectious Disease Products’, (2018) 73(3) FDLJ 361. See also Rutschman Ana Santos, ‘IP Preparedness for Outbreak Diseases’ (2018) 65 UCLA LR 1200.

6 Eric E. Johnson, and Theodore C. Bailey, ‘Urgent Legal Lessons from a Very Fast Problem: COVID‐19’ (2020) 73 SLR (forthcoming 2020) <https://ssrn.com/abstract=3567412>

7 Rimmer Matthew, ‘The Race to Patent the SARS Virus: The *TRIPS Agreement* and Access to Essential Medicines’ (2004) 5 MJIL.

8 Communia, Open Letter to WIPO: Intellectual Property and COVID‐19, 2020 <https://www.communia-association.org/2020/04/03/open-letter-wipo-intellectual-property-covid-19/>

9 Ana Santos Rutschman, ‘The Mosaic of Coronavirus Vaccine Development: Systemic Failures in Vaccine Innovation’ (2020) JIA (forthcoming); Saint Louis U. Legal Studies Research Paper No. 2020‐01 <https://ssrn.com/abstract=3559460 or https://doi.org/10.2139/ssrn.3559460> See also Ana Santos Rutschman, ‘The COVID‐19 Vaccine Race: Intellectual Property, Collaboration(s), Nationalism and Misinformation’ (2020) 64 WUJLP <https://ssrn.com/abstract=3656929>

10 One should note that TRIPS, for example, still provides for the compulsory protection of intellectual property (IP) rights by WTO member states. Such a global IP system has, in line with the suggestions of the Austrian School of Economics, been designed to induce research, entrepreneurial activity and provide incentives for innovative, wealth‐maximising behaviour (dynamic efficiency).

11 See, for example, Ana Santos Rutschman, ‘The Mosaic of Coronavirus Vaccine Development: Systemic Failures in Vaccine Innovation’ (2020) JIA (forthcoming); Saint Louis U. Legal Studies Research Paper No. 2020‐01.

12 See, for example, Kevin Bryan Jorge Lemus, and Guillermo Marshall, ‘Innovation During a Crisis: Evidence from COVID‐19’ (2020) <https://ssrn.com/abstract=3587973> or <https://doi.org/10.2139/ssrn.3587973>

13 For a synthesis of law and economics scholarship, see Gerrit De Geest, *Contract Law and Economics—Encyclopaedia of Law and Economics, Volume 6* (2nd edn, Edward Elgar Cheltenham, 2011). Also see R.A. Posner, *Economic Analysis of Law* (8th edn, Wolters Kluwer Law Publishers 2011).

14 Hence, there might not be any intellectual property rights issues arising over the most critical parts involved in COVID‐19 vaccine manufacturing.

15 Assuming that patents are filled and granted over a specific COVID‐19 vaccine and the manufacturing process thereoff only certain countries with a substantial pharma/generics presence, such as India and Korea, may posses the relevant infrastructure and human exertis to infirnge such patents, making such infringement unlikely.

16 Posner A. Richard, *Economic Analysis of Law* (9th edn, Wolters Kluwer 2014).

17 See North C. Douglas, *Institutions, Institutional Change and Economic Performance* (Cambridge University Press 1990); and North C. Douglas, *Understanding the Process of Economic Change* (Princeton University Press 2005).

18 Fox G. Harold, *Monopolies and Patents: A Study of the History and Future of the Patent Monopoly* (University of Toronto Press 1947).

19 Robert Cooter and Hand Bernd Schaefer, *Solomon's Knot: How Law can End the Poverty of Nations* (Princeton University Press 2012). See also North C. Douglas, *Institutions, Institutional Change and Economic Performance* (Cambridge University Press 1990); and North C. Douglas, *Understanding the Process of Economic Change* (Princeton University Press 2005).

20 McKaay Ejan, *Law and Economics for Civil Law Systems* (Edward Elgar 2013).

21 See, for example, Posner A. Richard, *Economic Analysis of Law* (8th edn, Wolters Kluwer 2011); and Cooter Robert and Thomas Ulen, *Law and Economics* (6th edn, Pearson 2016).

22 Cooter Robert and Thomas Ulen, *Law and Economics* (6th edn, Pearson 2016).

23 See, for example, McKaay Ejan, *Law and Economics for Civil Law Systems* (Edward Elgar 2013); Robert and Ulen (n 21).

24 Richard (n 16).

25 See Stefan Bechtold, Christopher J. Buccafrusco, and Christopher Jon, Sprigman, Innovation Heuristics: Experiments on Sequential Creativity in Intellectual Property’ (2015) ILJ (forthcoming); Cardozo Legal Studies Research Paper No. 475 <https://ssrn.com/abstract=2704107>

26 Ibid. See also Christopher J. Buccafusco and Christopher Jon Sprigman, ‘The Creativity Effect’ (2011) 78 UCLR 31; and Christopher J. Buccafusco and Christopher Jon Sprigman, ‘Valuing Intellectual Property: An Experiment’ (2010) 91 CLR.

27 Ibid. See also Eric E. Johnson, ‘Intellectual Property and the Incentive Fallacy’ (2011) 39 FSULR 623.

28 Ana Santos Rutschman, ‘The Intellectual Property of Vaccines: Takeaways from Recent Infectious Disease Outbreaks’ (2020) MLRO (forthcoming) <https://ssrn.com/abstract=3546368> or <https://doi.org/10.2139/ssrn.3546368>

29 For example, Zika was first discovered in 1947, but with no incentives to develop a drug, no one had a vaccine or a drug to stop the outbreak that occurred in 2015–2016. A very similar story happened with Ebola (which has been known since 1976), when a Canadian company already developed (and also patented in 2003) a promising vaccine and also finished a trial on animals, but did not continue to also test it on humans due to a lack of interest in the private sector, in 2014–2016, when cases of Ebola peaked, there was no approved drug, vaccine or treatment available in the market and there still was not until Ervebo became the first approved cure for Ebola in December 2019. The problem was and remains that there is funding only when an outbreak spikes. If only the incentives to find a vaccine for Ebola had been improved, not so many lives would been lost in the Ebola outbreak in 2014.

30 Ibid.

31 CEPI was founded in 2017 because of the recurring problem of lacking vaccines when needed; at the time, for Ebola. It is mostly funded by the governments of Norway, Japan and Germany, the Bill & Melinda Gates Foundation and the Welcome Trust, but in total it has about 70 investors. The CEPI's purpose is to make expensive vaccines accessible to lower‐income countries. They do this so by first funding investors and making them share clinical trial data, positive and negative results and, if the vaccine is suitable for the market, either a developer must return to the CEPI (back to the CEPI funding pool) all of the money put in their research or by giving up their IP rights and receiving only a proportionate amount of payment for vaccines. This is how they intend to make vaccines equally available even to lower‐income countries. Currently with COVID‐19, the CEPI was one of the first to respond in late December 2019 with its own CEPI funding pools; Ana Santos Rutschman, ‘The Intellectual Property of Vaccines: Takeaways from Recent Infectious Disease Outbreaks’ (2020) MLRO (forthcoming) <https://ssrn.com/abstract=3546368> or <https://doi.org/10.2139/ssrn.3546368>

32 Sam Halabi, Michelle Rourke, and Rebecca Katz, ‘The Law and Ethics of Data Sharing during Infectious Disease Emergencies’ (2019) JHCLP (forthcoming) <https://ssrn.com/abstract=3375089>

33 Ibid.

34 Ibid.

35 Eric E. Johnson and Theodore C. Bailey, ‘Urgent Legal Lessons from a Very Fast Problem: COVID‐19’ (2020). SLRO (forthcoming) <https://ssrn.com/abstract=3567412>

36 Ibid.

37 Of course, as they suggest, there is an agreement among some researchers to share all information they have discovered about COVID‐19 so far due to ethical reasons, but it is clear that there is a large number of researchers who are not driven by ethical reasons but mostly by money; Ibid.

38 Ibid.

39 They advocate a system of rewards for anyone who contributed any information which helped to develop a new vaccine. As they note, this would be harder to do in practice, but it is a way to encourage sharing important information and findings, which is crucial for saving time and many lives in this pandemic (namely, even if researchers want to act ethically and are willing to give up their patent rights by sharing their findings, they still need some money because research is very costly and researchers require money to fund it); ibid.

40 Jonathan J. Darrow and Michael Sinha, and Aaron S. Kesselheim, ‘When Markets Fail: Patents and Infectious Disease Products’ (2019) 73(3) FDLJ 361.

41 Also, a vaccine for a disease can be much less profitable than a regular treatment, which can last a lifetime for some people and brings much more money than a vaccine, which can be given in maximum of three doses. Hence, there is some legislation that helps keep incentives for developers, such as: Priority Review Vouchers, the GAIN Act, the ‘Limited Population Pathway’ Act, and also Non‐profit Organisations and Government Funding of Basic Research; Ibid.

42 Magnusson discusses the TRIPS Agreement, the Patent Act of Zimbabwe, the Industrial Property Law of Brazil and the already existing Medicines Patent pool established by the International Drug Purchase Facility in 2006 to help in negotiating over licence agreements in low‐ and middle‐income countries for HIV. He also discusses the Benefit‐sharing provisions applicable to commercial entities receiving influenza viruses under the Pandemic Influenza Preparedness Framework; Roger Magnusson, *Advancing the Right to Health: The Vital Role of Law* (World Health Organization, 2017); Sydney Law School Research Paper No. 17/43 <https://ssrn.com/abstract=2979054>

43 Dana Beldiman, Patent Chokepoints in the Influenza‐Related Medicines Industry: Can Patent Pools Provide Balanced Access? (2012) 15 TJTIP 31.

44 Ibid.

45 Also, how it is hard to develop a certain product (e.g., a vaccine), when multiple owners hold a patent for the components and how it consumes very crucial time and resources. Once someone has developed a vaccine/treatment and successfully patented it, its downfall is that others cannot or face many unnecessary issues if they want to improve it. Supposedly, there are also many patented inventions that remain sitting on shelves because it is too costly and risky to put them in the market; ibid.

46 Frank Tietze, Pratheeba Vimalnath, Leonidas Aristodemou, and Jenny Molloy, *Crisis‐Critical Intellectual Property: Findings from the COVID‐19 Pandemic* (2020). Centre for Technology Management Working Paper Series, No. 2, April 2020 <https://ssrn.com/abstract=3569282> or <https://doi.org/10.2139/ssrn.3569282>

47 Ibid.

48 Ibid.

49 Qiwei Xue and Lisa Larrimore Ouellette, ‘Innovation Policy and the Market for Vaccines’ (2020) JLB (forthcoming); Stanford Law and Economics Olin Working Paper No. 549 <https://ssrn.com/abstract=3595756>

50 ‘The preventative and durable aspects of vaccines generate substantial social benefits, but policymakers should recognize that these features also reduce incentives to develop vaccines in the first place—particularly when these benefits lead to political pressure for low vaccine prices’; Ibid.

51 Santos Rutschman states there are supposedly a couple of vaccines already on trial and they are to come to market in a record time, in a few months, instead of about 1 year. In mid‐March, Chile already passed a resolution allowing the government to issue compulsory licences for anything related to COVID‐19 treatment; Ana Santos Rutschman, ‘The Mosaic of Coronavirus Vaccine Development: Systemic Failures in Vaccine Innovation’ (2020) JIA (forthcoming); Saint Louis U. Legal Studies Research Paper No. 2020‐01. See also Santos Rutschman (n 4).

52 Karen Walsh, Andrea Wallace, Mathilde Pavis, Olszowy Natalie, Griffin James and Naomi Hawkins, ‘Intellectual Property Rights and Access in Crisis’ (2021) 52 IRIPCL 379. See also Hyo Yoon Kang, ‘COVID‐19: Who Is the “Public” in IP Law That Defines Its Public Purpose?’ (2021) Science, The Wire <https://science.thewire.in/health/covid-19-who-is-the-public-in-ip-law-that-defines-its-public-purpose/>

53 McMahon calls for a reconsideration of the role of bioethics within patent law is needed and long overdue, and COVID‐19 provides us with an important opportunity to reframe this relationship between health and patents; McMahon Aisling, ‘Covid‐19, Patents & Healthcare: The Need for A (Bio)ethics Space within Patent Law’ (2020) JME <https://blogs.bmj.com/medical-ethics/2020/04/16/covid-19-patents-healthcare-the-need-for-a-bioethics-space-within-patent-law/>. See also Sell K. Susan, ‘What COVID‐19 Reveals About Twenty‐First Century Capitalism: Adversity and Opportunity’ (2020) 63 Development 150; and Amaki Vanna, ‘On Intellectual Property Rights, Access to Medicines and Vaccine Imperialism’ (2021) 32 TWAILR.

54 Correa M. Carlos, ‘Intellectual Property Rights and Inequalities in Health Outcomes’ in Ronald Labonté, Ted Schrecker, Corinne Packer, and Vivien Runnels (eds.), *Globalization and Health: Pathways, Evidence and Policy* (Routledge 2009). See also Chapman R. Audrey and Salil D. Benegal, ‘Globalization and the Right to Health’, in Lanse Minkler (ed.), *The State of Economic and Social Human Rights* (Cambridge University Press, 2013); and Salazar Silvia, *Intellectual Property and the Right to Health* (WIPO‐UNCHR/IP/PNL/98/3, 1998); and World Health Organization, ‘Public health, Innovation and Intellectual Property Rights’ (Report of the Commission on Intellectual Property Rights, Innovation and Public Health 2006). However, Dreyfuss argues that the characterisation of patent rights as human rights is not only wrong, but has also unfortunate pragmatic consequences (leading to unpredictable decision‐making and less conducive environment for investing time and money in intellectual efforts) since it is harder to make a case for intruding on patent prerogatives when they are characterised as human rights than when they are justified on utilitarian grounds; Dreyfuss Rochelle Cooper, ‘Patents and Human Rights: Where is the Paradox?’ in Willem Grosheide (ed.), *Intellectual Property and Human Rights: A Paradox* (Edward Elgar 2010).

55 Light W. Donald, ‘Addressing Health Care Disparities: A Radical Perspective and Proposal’ (2020) 5 FS 29. Also Baker suggest that current IP law might be the cause of inequality and that IP policy should take into account the inequality debate; Baker Dean, ‘Is Intellectual Property the Root of All Evil? Patents, Copyrights, and Inequality’ (Working paper, University of Utah, Department of Economics 2018).

56 Garrett Hardin, ‘The Tragedy of the Commons’ (1968) 162 Science 124.

57 See, for example, Francesco Parisi, and Ben Depoorter, *The Law and Economics of the Anticommons: Issues in Property Law* (Edward Elgar 2020); Leitzel Jim, *Concepts in Law and Economics* (Oxford University Press 2015) pp. 20‐21.

58 Garrett Hardin, ‘The Tragedy of the Commons’ (1968) 162 Science 124. The specific theory of common property resource management is seminally treated in H. Scott Gordon, ‘The Economic Theory of a Common Property Resource: The Fishery’ (1954) 62 JPE 24, and in Anthony Scott, ‘The Fishery: The Objectives of Sole Ownership’ (1955) 63 JPE 116. Elinor Ostrom, Governing the Commons: The Evolution of Institutions for Collective Action (1990), examines alternative institutional means through which common property resources have in fact been managed.

59 ‘There might be an underuse of valuable asset because control rights are so dispersed that it is hard for all owners of these rights to agree to specific use of the asset. As a result, the value of the asset in practice will be greatly diminished or eliminated;’ Leitzel, *supra* note 69, p. 164.

60 Michael A. Heller, ‘The Tragedy of the Anti‐commons: Property in the Transition From Marx To Markets’ (1998) 111 HLR 621.

61 Leitzel, supra note 69, at p. 23. See also F Parisi and B Depoorter, ‘Commons and Anticommons’ in CK Rowley and F Schneider (eds) *The Encyclopedia of Public Choice* (Springer 2004).

62 Carl Shapiro, ‘Navigating the Patent Thicket: Cross Licenses, Patent Pools, and Standard Setting’ (2001) 1 IPE 119.

63 They suggest that ‘privatization of biomedical research must be more carefully deployed to sustain both upstream research and downstream product development’… and that ‘more intellectual property rights may lead paradoxically to fewer useful products for improving human health’; MA Heller and RS Eisenberg, ‘Can Patents Deter Innovation? The Anticommons in Biomedical Research’ (1998) 280 Science 698.

64 If the effectiveness of secrecy is an important factor in inventors' patenting decisions, scientific breakthroughs, which facilitate reverse‐engineering, should increase inventors' propensity to patent; Petra Moser, ‘Innovation without Patents: Evidence from World's Fairs’ (2012) 55(1) TJLE 43.

65 Ibid.

66 Ibid.

67 Sven Vanneste, Alain Van Hiel, Francesco Parisi, and Ben Depoorter, ‘From “Tragedy” to “Disaster”: Welfare Effects of Commons and Anticommons Dilemmas’ (2006) 26 IRLE 104. See also Francesco Parisi, Ben Depoorter, and Norbert Schulz, ‘Duality in Property: Commons and Anticommons’ (2005) 25(4) IRLE.

68 Ibid.

69 As they report, these results were obtained with ‘different methodologies (i.e., lab experiment vs. scenario experiment), different research designs (i.e., simultaneous presentation of the two types of dilemma resulting in a within‐subjects design vs. presentation of different dilemmas in a between‐subjects design), and different modalities (e.g., free bidding vs. the use of a pay‐off scheme), attesting to the stability of these findings and their broad generality’; Vanneste et al., Ibid.

70 K Maskus, *Intellectual Property Rights in the Global Economy* (Petersen Institute for International Economics 2000). Petersen Institute for International Economics, Washington in EU Bilateral Trade Agreements and Intellectual Property: For Better or Worse?, 2014, p. 170.

71 See, for example, Justine Pila and Paul Torremans, *European Intellectual Property Law* (Oxford University Press 2016).

72 Ibid., p. 123.

73 European Patent Convention 1973/2000.

74 Pila and Torremans, *supra* note 88, p. 124.

75 ‘… firms are filing several patents in respect of a single medicine, such as product patent for the active substance comprising the medicine, a process patent for the method of producing the medicine and a purpose‐limited product patent for the medicine when used either for a different purpose of for the same purpose but via a different dosage regime’; ibid.

76 EU Commission, ‘Pharmaceutical Sector Inquiry: Final Report’ Brussels, 8 July 2009.

77 Ibid.

78 Patent Cooperation Treaty, Made in Washington on 19 June 1970, amended on 28 September 1979, modified on 3 February 1984 and on 3 October 2001.

79 Agreement on the Trade‐Related Aspects of Intellectual Property Rights, WTO, New York, 1 January 1995.

80 Compulsory licensing enables governments (WTO members) to allow firms, legal entities or individuals to produce a patented product or process without the consent of the patent owner or plans to use the patent‐protected invention itself. This possibility enables flexibility in the field of patent protection and is included in the WTO's agreement on intellectual property—the TRIPS; WTO, ‘Compulsory licensing of pharmaceuticals and TRIPS’ 2020 <https://www.wto.org/english/tratop_e/trips_e/public_health_faq_e.htm>

81 However, it has to be emphasised that in instances of ‘national emergencies’, ‘other circumstances of extreme urgency’ or ‘public noncommercial use’ (or ‘government use’) or anticompetitive practices, there is no need to try first for a voluntary licence; Ibid.

82 Article 31 of the TRIPS provides that: ‘(b) such use may only be permitted if, before such use, the proposed user has made efforts to obtain authorisation from the right holder on reasonable commercial terms and conditions and that such efforts have not been successful within a reasonable period of time. This requirement may be waived by a Member in the case of a national emergency or other circumstances of extreme urgency or in cases of public noncommercial use. In situations of national emergency or other circumstances of extreme urgency, the right holder shall, nevertheless, be notified as soon as reasonably practicable. In the case of public noncommercial use, where the government or contractor, without making a patent search, knows or has demonstrable grounds to know that a valid patent is or will be used by or for the government, the right holder shall be informed promptly; (c) the scope and duration of such use shall be limited to the purpose for which it was authorized, and in the case of semiconductor technology shall only be for public noncommercial use or to remedy a practice determined after judicial or administrative process to be anticompetitive; (d) such use shall be nonexclusive; (g) authorisation for such use shall be liable, subject to adequate protection of the legitimate interests of the persons so authorized, to be terminated if and when the circumstances which led to it cease to exist and are unlikely to recur. The competent authority shall have the authority to review, upon motivated request, the continued existence of these circumstances; (h) the right holder shall be paid adequate remuneration in the circumstances of each case, taking into account the economic value of the authorization’.

83 Article 31 of the TRIPS.

84 One should note that the Doha Declaration on TRIPS and Public Health confirms that countries are free to determine the grounds for granting compulsory licences, and to determine what constitutes a national emergency; WTO, ‘Compulsory licensing of pharmaceuticals and TRIPS’ 2020 <https://www.wto.org/english/tratop_e/trips_e/public_health_faq_e.htm>

85 Namely, initially it meant that it was impossible to manufacture a patented drug under compulsory licence in Country A, with the intention of exporting the drug to Country B; Lionel Bentley and Brand Sherman, *Intellectual Property Law* (3rd ed., Oxford University Press 2009), pp. 352–353.

86 WTO, Implementation of para 6 of the Doha Declaration on the TRIPS Agreement and Public Health, WT/L/540, 1 Sept. 2003.

87 The WTO suggests that the amended rules merely create the legal pathway, but countries have to make use of it. Because it concerns production for export, those countries seeking to export under the system may need to amend their laws to ensure that such production is permissible under compulsory licences; Kampf, Roger, ‘Special compulsory licences for export of medicines: Key features of WTO members' implementing legislation’, WTO, 2015. See also Carlos M. Correa and A. Yusuf Abdulqawi, *Intellectual Property and International Trade Trips Agreement* (Edward Elgar 2008); and Carlos M. Correa, *Trade Related Aspects of Intellectual Property Rights: A Commentary on the TRIPS Agreement (Oxford Commentaries on International Law)* (Oxford University Press 2020).

88 Bentley and Sherman, *supra* note 97, p. 354. See also by Antony Taubman, Hannu Wager, and Jayashree Watal, ‘A Handbook on the WTO TRIPS Agreement’ (Cambridge University Press 2012).

89 Dorothy R. Auth, ‘COVID‐19 Update: Patent Rights in the COVID‐19 Pandemic: How Will Industries and Governments Respond?’ (2020) 10 NLR 260.

90 John R. Thomas, ‘March‐In Rights Under the Bayh‐Dole Act’ (2016) CRS 7‐5700.

91 The Bayh–Dole Act actually allows academic scientists who received US government funds for research to patent their findings and license them to private companies which have the resources to turn the insights into tangible products. The Bayh–Dole Act of 1980 allowed the private patenting of inventions discovered with federally funded research. Congress balanced that significant benefit to private entities by empowering the government to ‘march in’ and grant a licence to another manufacturer when the licence holder has not made the invention available to the public on reasonable terms. Bayh–Dole also allows a march‐in when necessary to alleviate health or safety needs.

92 On the other hand, Penman and Quigley argue that the current medicines pricing and access crisis, highlighted by the inaccessibility of an effective prostate cancer drug discovered with government funding, calls for US agencies to finally fulfil the terms of the Act; Jennifer Penman and Fran Quigley, ‘Better Late than Never: How the US Government Can and Should Use Bayh‐Dole March‐In Rights to Respond to the Medicines Access Crisis’ (2017) 53(fall) WLR.

93 Fred Reinhart, ‘Exercising Bayh‐Dole march‐in rights would handicap Covid‐19 innovation’, STAT 2020 <https://www.statnews.com/2020/05/04/bayh-dole-march-in-rights-handicap-covid-19-innovation/>

94 The ‘conventional view’ of the patent system as a device that enables an inventor to capture the returns from their investment in the invention will be called the reward theory. For a synthesis, see Peter S. Menell and Suzanne Scotchmer, ‘Intellectual Property Law’, in Polinsky A. Mitchell and Steven Shavell (eds.), *Handbook of Law and Economics* (Vol. 2, North‐Holland 2007), pp. 1473‐1571.

95 The US pharmaceutical company Merck criticised Brazil's licensing of its HIV drug efavirenz as an ‘expropriation of intellectual property’ that may in the long run ‘hurt patients who require new life‐saving therapies’ (Intellectual Property Watch, 7 May 2007).

96 Third parties can then search for ways to increase the value of the patent and, when they find it, force the owner to license the patent at the regulated rate; Kitch W. Edmund, ‘The Nature and the Function of the Patent System’ (1977) 20 JLE 2, 265.

97 Reinhart, *supra* note 110.

98 ‘And that would inevitably slow the development and reduce the availability of new treatments and vaccines, hurting patients everywhere. Affordability is a goal all parties share, but without a pipeline of novel products, pricing becomes irrelevant’, ibid.

99 Baten, Bianchi and Moser argue that this is primarily due to the fact that contemporary settings offer little exogenous variation in compulsory licensing. Yet, they address this empirical challenge by exploiting an event of compulsory licensing as a result of World War I when the US Trading with the Enemy Act made all German‐owned patents available for licensing to US firms. Their analysis of the firm‐level data of German patents shows that compulsory licensing was associated with a 30% increase in invention by German firms whose inventions were licensed; chiefly because contemporary settings offer little exogenous variation in compulsory licensing; Joerg Baten, Nicola Bianchi, and Petra Moser, ‘Compulsory licensing and innovation—Historical evidence from German patents after WWI’ (2017) 126 JDE 231. See also Richard Rozek, ‘The Effects of Compulsory Licensing on Innovation and Access to Health Care’ (2005) 6 JWIP, 889.

100 Eric W. Bond and Kamal Sagi, ‘Compulsory licensing, price controls, and access to patented foreign products’ (2014) 109 JDE 217. Moreover, Kyung‐Bok argues that compulsory licences might have a new role in current patent systems: compulsory licensing could be a potential alternative or complement to ensure access to medicines in health systems through manufacturing and exporting patented pharmaceuticals; Kyung‐Bok Son, ‘Importance of the intellectual property system in attempting compulsory licensing of pharmaceuticals: a cross‐sectional analysis’ (2019) 15 GH 42. See also Colleen Chien, ‘Cheap Drugs at What Price to Innovation: Does the Compulsory Licensing of Pharmaceuticals Hurt Innovation?’ 2003 BTLJ. Whereas Mozer and Voena, while assessing the impact of compulsory licences on the innovative activity of the country that invoked such a compulsory licence, show that compulsory licensing increased domestic invention by 20%; Moser Petra and Alessandra Voena, ‘Compulsory Licensing: Evidence from the Trading with the Enemy Act’ (2012) 102 AER 396.

101 Chien Colleen, ‘Cheap Drugs at What Price to Innovation: Does the Compulsory Licensing of Pharmaceuticals Hurt Innovation’ (2003) 18 BTLJ 853.

102 ‘On the other hand, if more companies like AbbVie take a more socially conscious approach, there may not be need for government intervention in terms of compulsory patent licenses’, Auth, *supra* note 106.

103 Ibid.

104 ‘AbbVie's bold stance would allow generic versions of Kaletra to be made by others without fear of repercussion based on patent infringement and would also allow countries to purchase generic versions of Kaletra if it is found effective in treating COVID‐19, and would help alleviate possible drug supply shortages’, Auth, ibid.

105 Analytically speaking, such a prospect of the governmental violation of IP rights should be seen as a cause of uncertainty and hence a source of direct and indirect costs.

106 Auth, *supra* note 106.

107 Benjamin Tham, and Mark James Findlay, ‘COVID‐19 Vaccine Research, Development, Regulation and Access’ (2020) SMU Centre for AI & Data Governance Research Paper (forthcoming) <https://ssrn.com/abstract=3640153>

108 Shavell Steven, ‘On optimal legal change, past behaviour and grandfathering’, Shavell, Steven, On Optimal Legal Change, Past Behavior, and Grandfathering (October 2007). NBER Working Paper No. w13563 <https://ssrn.com/abstract=1024977>

109 According to Shavell, the reasons are twofold: adjusting to a new legal rule often involves costs; and the social benefits of change are frequently merely incremental, only in addition to those of past compliance; ibid.

110 Ibid. See also Gersen E. Jacob and Eric A. Posner, ‘Timing Rules and Legal Institutions’ (2007) 121 HLR 2, 543.

111 Cafaggi Fabrizio, Antonio Nicita, and Ugo Pagano, *Legal orderings and economic institutions* (Routledge 2007).

112 See, for example, Lawrence E. Blume and Daniel L. Rubinfeld, ‘The Dynamics of the Legal Process’ (1982) 11 JLS 2.

113 Francesco Parisi, Vincy Fon and Nita Ghei, ‘The Value of Waiting in Law‐making’ (2004) 18(2) EJLE 131; See also Parisi and Ghei, supra note 69.

114 Ibid.

115 Ibid.

116 They offer as an example legislating in a field dominated by rapidly changing technology, such as the Internet, which carries a high value of waiting. The field is growing in importance, and legislating today may leave the system with inefficient laws for the future. On the other hand, ‘the greater the short‐term benefits of legal intervention, the lower is the net value of waiting. Thus, for example, environmental legislation may have substantial immediate benefits, and irreversible losses that might result from delay. This reduces the value of waiting. Thus, the greater the immediate value of legal innovation, the more likely is immediate adoption of the new rule’, ibid.

117 March‐in‐rights distort incentives to innovate and the enforcement of such march‐in rights might be viewed as a source of dynamic inefficiencies, increased transaction costs and greater uncertainty (for innovators and research activity).

118 Bryan et al. (n 12) <https://ssrn.com/abstract=3587973> or <https://doi.org/10.2139/ssrn.3587973>

119 Of course, the ‘demand side” of the market encompasses also many additional external or internal factors (both push and pull) that may affect veccine development & innovation paterns.

120 The SARS outbreak in total caused 916 deaths and infected 8422 people. See WHO, Summary table of SARS cases by country, 1 November 2002–7 August 2003, WHO, 2003.

121 Yet, when a pathogen managed to infect the entire planet or entire continents then the demand side of the market combined with the IP‐incentive stream results in real, world‐wide competition.

122 See, for example, Rutschman, who states that ‘apart from historically ingrained pervasiveness of patent regimes in contemporary innovation structure there is no particular reason why vaccine R&D should be considered to be best incentivized through intellectual property incentives’, supra note 6. See also Ana Santos Rutschman, ‘IP preparedness for outbreak diseases’ (2018) 65 UCLA LR 1200; and Ana Santos Rutschman, ‘The intellectual property of vaccines: takeaways from recent infectious disease outbreaks’ (2020) 118 MLRO 170.

123 As argued, the cause of such a slow reaction of the private sector to engage in vaccine research and development and large pharmaceutical companies to start developing a vaccine were experienced in previous infectious disease outbreaks, such as Ebola, Zika and SARS, in all of which the industry interest in a vaccine came to a halt when the outbreak slowed down. What is more, even when funding for expedited research and development became available, they at first remained unappealing to the majority of vaccine developers due to the unprofitability of the infectious disease market; Xue and Ouellette (n 49) <https://ssrn.com/abstract=3595756>

124 For example, Schmookler argues that ‘invention is largely and economic activity, which like other economic activities, is pursued for gain’ and that the ‘amount of invention is governed by the extent of the market’, Jacob Schmookler, *Invention and Economic Growth* (Harvard University Press 1966). See also Michael Kremer, ‘Pharmaceuticals and the Developing World’ (2002) 16 JEP 67; Fiona Scott Morton, ‘Entry decisions in the generic industry’ (1999) 30 RJ 421; Ariel Pakes and Mark Schankerman, ‘An explanation into the determinants of research intensity’, in Griliches Zvi (ed.), *R&D Patents and Productivity* (Chicago University Press 1984); and Zvi Griliches, ‘Hybrid Corn: an exploration in the economics of technological change’, (1957) 25 Econometrica 501.

125 Daron Acemoglu and Joshua Linn, ‘Market size and innovation: theory and evidence from the pharmaceutical industry’ (2004) 119 QJE 3, 1049.

126 Their results show an important effect of potential market size on pharmaceutical innovation; Ibid, p. 1051.

127 Edwin Mansfield, ‘Patents and Innovation: An Empirical Study’ (1986) 32 MS 2, 173.

128 Type I error, also known as a ‘false positive’, is the error of rejecting a null hypothesis when it is actually true. In other words, this is the error of accepting an alternative hypothesis (the real hypothesis of interest) when the results can be attributed to chance. Plainly speaking, it occurs when we are observing a difference when in truth there is none (more specifically, no statistically significant difference). Type II error, also known as a ‘false negative’, is the error of not rejecting a null hypothesis when the alternative hypothesis is the true state of nature. Namely, this is the error of failing to accept an alternative hypothesis when you do not have adequate justification. Put simply, it occurs when we fail to observe a difference when in truth one exists.

129 Such Type II errors can result in the loss of significant benefits to society when the sale of vaccines that are safe and effective is delayed.

130 See, for example, Michael D. Intriligator, ‘Drug Evaluations: Type I versus Type II Errors’, UCLA: Research Program in Pharmaceutical Economics and Policy, 1996 <https://escholarship.org/uc/item/5fg9n284>

131 One involving the loss function that incorporates the benefits and losses of both types of errors.

132 For example, such an approach would strike a balance between the two possible errors (taking explicit account of the consequences of not approving safe and effective vaccines in terms of delays of their availability. Moreover, some of the additional clinical testing might, due to diminishing returns of additional testing, represent the social deadwieght loss and hence has little social value; ibid. See also Peltzman Sam, ‘The Benefits and Costs of new Drug Regulation’, in Richard Landau (ed.), *Regulating New Drugs* (University of Chicago Press 1973); McGuire Thomas, Nelson Richard, and Thomas Spavins, ‘An Evaluation of Consumer Protection Legislation: The 1962 Drug Amendments’ (1974) 83 JPE 655; and Viscusi W. Kip, *Fatal Tradeoffs: Public and Private Responsibilities for Risk* (Oxford University Press 1992).

133 Xue and Ouellette (n 49).

134 Thampapillai argues that due to its voluntary character mostly only smaller developers, which cannot afford to be directly involved in drug trials and manufacturing, are participating and bigger medical companies, which can afford to carry out the last stages such as manufacturing and final drug trials by themselves, are keeping the useful information private; Thampapillai, supra note 126. See also Santos (n 9). Washington University Journal of Law and Policy, Vol. 64, 2020 <https://ssrn.com/abstract=3656929>

135 That is where compulsory licensing becomes the solution—it does not discourage vaccine developers as they can claim royalties from other countries or buyers in general that can afford vaccine at the given price and it also gives the right to countries in development to recreate the vaccine without paying any patent royalties for a much lower price.

136 Namely, Tham et al. argue that the pharmaceutical firm that should bring its vaccine to the market might refrain from patenting because it knows that if anyone wanted to recreate its vaccine by reverse engineering the process of development and necessary trials would take too long to bring the recreated vaccine to the market in time to attract any potential buyers. This is where the compulsory licensing cannot help with the supply of vaccines also to poorer countries and global health equality is not accomplished; Tham, Benjamin and Findlay, Mark James, COVID‐19 Vaccine Research, Development, Regulation and Access (30 June 2020). SMU Centre for AI & Data Governance Research Paper (forthcoming) <https://ssrn.com/abstract=3640153>

137 Tietze et al, supra note 25.

138 Kremer et al. suggest that this would be economically equivalent to putting high taxes on a narrow base, which can be an inefficient way of raising revenue, and if there are significant out‐of‐pocket payments among patients that would also raise equity concerns; Michael Kremer, Towse Adrian, and Heidi Williams, Briefing Note on Advance Purchase Commitments (UK Department for International Development 2005).

139 Gavin Yamey, Marco Schäferhoff, Muhammad Pate, Mukesh Chawla, Kent Ranson, Richard Hatchett, and Richard Wilder, *Funding the Development and Manufacturing of COVID‐19 Vaccines*. Background paper for the World Bank/CEPI financing COVID‐19 vaccine development consultation on 20 February 2020. The Center for Policy Impact in Global Health, Duke Global Working Paper Series No. 20, March 2020, <https://ssrn.com/abstract=3575660> or <https://doi.org/10.2139/ssrn.3575660>

140 Yamey, chäferhoff, Pate, Chawla, Ranson, Hatchett and Wilder, *supra* note 139.

141 Qiwei and Ouellette, *supra* note 124.

142 Ibid.

143 This would cause herd immunity, which would also benefit the government by helping to lower the toll on the national economy: ibid.

144 The literature shows that up to USD 2 billion is needed to accelerate the development of, scale up, and prepare to roll out vaccines against COVID‐19; Yamey and Others, ‘Funding the development and manufacturing of COVID‐19 vaccines’, Duke Global Working paper 20, 2020.

145 At the moment, out of such a large number of developers, only a couple of vaccines are in phase‐three stages or were early approved like in China and Russia, respectively. Most of those vaccines in third‐phase trials also have an advance purchase agreement with the EU, the United States, Canada, the United Kingdom, Australia, and Switzerland. These vaccine efforts are also mostly led by larger pharmaceutical companies.

146 See C Zimmer, S Wee, and J Corum, *Coronavirus Vaccine Tracker* [online] Nytimes.com. (2020) <https://www.nytimes.com/interactive/2020/science/coronavirus-vaccine-tracker.html> accessed 6 October 2020.

147 Michael Kremer, Towse Adrian, and Heidi Williams, *Briefing Note on Advance Purchase Commitments* (UK Department for International Development 2005). See also Adrian Towse, Eric Keuffel, Hannah E. Kettler, and David B. Ridley, *Drugs and Vaccines for Developing Countries* (Office of Health Economics 2011).

148 Ibid.

149 H Kettler, ‘The Biotechnology Industry and Orphan Drug Incentive: A Win‐Win Strategy for Europe?’ (2000) 7(1) JCB 62.

150 Kettler E. Hannah and Sonja Marjanovic, ‘Engaging Biotechnology Companies in the Development of Innovative Solutions for Diseases of Poverty’ (2004) 3 NRDD 2, 171. In addition, Finkelstein finds a strong positive impact of such market‐increasing policies on private sector R&D activity on affected vaccines; Finkelstein (2004) ‘Static and Dynamic Effects of Health Policy: Evidence From the Vaccine Industry’ (2004) 119(2) QJE 527.

151 In addition, some types of pull mechanisms may disproportionately favour one type of firm (e.g., large pharmaceutical firms), which may not be the optimal structure in terms of innovation incentives; A Towse and H Kettler, ‘A Review of IP and Non‐IP Incentives for R&D for Diseases of Poverty. What Type of Innovation is Required and How Can We Incentivise the Private Sector to Deliver It?’ (2005) Working Paper for the Commission on Intellectual Property Rights, Innovation and Public Health (CIPIH) (World Health Organization 2005).

152 See Bryan et al. (n 12).

153 Ibid.

154 In addition, it may be a multiyear commitment and with advance purchase commitments sponsors need only specify what they are looking for in a desired product, but not specify how the goal should be achieved.

155 European Commission—European Commission. 2020. *Press Corner*. [online] <https://ec.europa.eu/commission/presscorner/detail/en/ip_20_1438> accessed 16 September 2020.

156 N Price, R Sachs, J.S. Sherkow, and Larrimore Ouellette, *COVID‐19 Vaccine Advance Purchases Explained | Bill of Health*. [online] Bill of Health (2020) <https://blog.petrieflom.law.harvard.edu/2020/08/11/covid19-vaccine-advance-purchases-explained/> accessed 16 September 2020.

157 Kremer et al. also suggest that, if structured correctly, advance purchase commitments can also facilitate access to these technologies if and when they are developed. Namely, in the ‘short‐term, access is facilitated through donor purchasers at the higher, prespecified purchase price. In the long‐term, financially sustainable access to these technologies is facilitated through the contract provision which requires developers to commit to drop the price to a low level (close to marginal cost) after all high‐price purchases have been made’; Kremer et al., *supra* note 152, p. 24. For a critical view on advance purchase agreements, see for example, A Farlow, D Light, R Mahoney, and R Widdus, ‘Concerns Regarding the Center for Global Development Report “Making Markets for Vaccines”’, Submission to the CIPIH, 29 April 2005.

158 Ibid.

159 Ibid. See also Ernst R. Bernd and Others, ‘Advance Market Commitments For Vaccines Against Neglected Diseases: Estimating Costs And Effectiveness’ (2007) 16 HE 5, 491.

160 See, for example, M Bennett, and P Collins, ‘The Law and Economics of Information Sharing: The Good, the Bad and the Ugly’ (2010) 6(2) ECJ, 311; A Capobianco, ‘Information Exchange Under EC Competition Law’(2004) 41(5) CMLR 1247; A. Capobianco, *Information Exchanges Between Competitors under Competition Law* (DAF/COMP (2010)26: OECD 2010); O Odudu, ‘Indirect Information Exchange: The Constituent Elements of Hub and Spoke Collusion’ (2011) 7(2) ECJ 205.

161 Council Regulation no. 1/2003 on the implementation of the rules of competition laid down in Articles 101 and 102 of the TFEU (the ‘Modernisation Regulation’). The new Council Regulation, which came into effect on 1 May 2004, replaces Council Regulation No. 17 which had been in force for over 40 years and been the key to the enforcement of Community competition law (for a synthesis, see Müller, 2004). New Regulation No. 1/2003 thus replaces the centralised notification and authorisation system with an enforcement system based on the direct application of Articles 101 and 102.

162 The horizontal sharing of knowledge (information), enhancement of information flows between undertakings, and co‐operation appear to be fundamental for creating the dynamic evolution of markets, improving cost‐efficient processes and enhancing social welfare.

163 Mitja Kovac and Patricia Kotnik, ‘Self‐Assessment System: Detrimental Effects On Entrepreneurial And Innovative Activity’ (2014) 5(1) JARLE 23.

